# Retrospective Robot-Measured Upper Limb Kinematic Data From Stroke Patients Are Novel Biomarkers

**DOI:** 10.3389/fneur.2021.803901

**Published:** 2021-12-21

**Authors:** Michela Goffredo, Sanaz Pournajaf, Stefania Proietti, Annalisa Gison, Federico Posteraro, Marco Franceschini

**Affiliations:** ^1^Department of Neurological and Rehabilitation Sciences, IRCCS San Raffaele Roma, Rome, Italy; ^2^Rehabilitation Department, Versilia Hospital, Azienda Unità Sanitaria Locale (AUSL) Northwest Tuscany, Camaiore, Italy; ^3^Department of Human Sciences and Promotion of the Quality of Life, San Raffaele University, Rome, Italy

**Keywords:** robot-assisted therapy, stroke rehabilitation, motor recovery, upper extremity, kinematics

## Abstract

**Background:** The efficacy of upper-limb Robot-assisted Therapy (ulRT) in stroke subjects is well-established. The robot-measured kinematic data can assess the biomechanical changes induced by ulRT and the progress of patient over time. However, literature on the analysis of pre-treatment kinematic parameters as predictive biomarkers of upper limb recovery is limited.

**Objective:** The aim of this study was to calculate pre-treatment kinematic parameters from point-to-point reaching movements in different directions and to identify biomarkers of upper-limb motor recovery in subacute stroke subjects after ulRT.

**Methods:** An observational retrospective study was conducted on 66 subacute stroke subjects who underwent ulRT with an end-effector robot. Kinematic parameters were calculated from the robot-measured trajectories during movements in different directions. A Generalized Linear Model (GLM) was applied considering the post-treatment Upper Limb Motricity Index and the kinematic parameters (from demanding directions of movement) as dependent variables, and the pre-treatment kinematic parameters as independent variables.

**Results:** A subset of kinematic parameters significantly predicted the motor impairment after ulRT: the accuracy in adduction and internal rotation movements of the shoulder was the major predictor of post-treatment Upper Limb Motricity Index. The post-treatment kinematic parameters of the most demanding directions of movement significantly depended on the ability to execute elbow flexion-extension and abduction and external rotation movements of the shoulder at baseline.

**Conclusions:** The multidirectional analysis of robot-measured kinematic data predicts motor recovery in subacute stroke survivors and paves the way in identifying subjects who may benefit more from ulRT.

## Introduction

More than 70% of stroke survivors suffer from upper limb impairment and from a kind of disability in the activities of daily living (1, 2). For these reasons, the recovery of the upper limb motor function and the reintegration into the real-life context have always been the main goals of post-stroke rehabilitation (3, 4). A Cochrane review of 2014 showed that any approach of high dose physical rehabilitation is more effective than usual care in improving motor functions (5). This outcome has been subsequently confirmed by Mehrholz et al. who reviewed the literature on the efficacy of upper limb Robot-assisted Therapy (ulRT) in improving activities of daily living, arm function, and arm muscle strength (6). The efficacy (7–11), acceptability (12), safety (13), and cost-effectiveness (14) of ulRT in stroke patients are well-established in literature.

Traditionally, the effects of ulRT are reported by using standardized clinical assessments [such as, the upper extremity subscale of the Fugl-Meyer Assessment (15), the upper limb Motricity Index (16, 17), or the Action Research Arm Test (18)] and biomechanical measurements of upper limb movements (19–22). Specifically, a multiplicity of kinematic parameters has been applied for upper limb evaluations, such as movement accuracy, speed, and smoothness, and some of them have been correlated to the clinical outcome measures (21, 23). Interestingly, a number of publications analyzed the Robot Measured Kinematic (RMK) data (i.e., the trajectories for movements) registered by the robot (20, 24–28) assessing the biomechanical changes induced by ulRT, and thus the patient progress, in terms of motor control and coordination. Moreover, these robotic measurements allow monitoring the time course of motor recovery during ulRT (24–27), showing that it is movement direction-dependent (20, 28). The robot-measured data have been processed not only for assessing the efficacy of ulRT, but also for predicting the clinical scales (29–31). Krebs et al. found that measurements of kinematics and kinetics recorded by a robot may predict the clinical outcomes registered on a given day (29), thus suggesting that the robotic measurements can be biomarkers of motor impairment. These findings were confirmed by Grimm et al. who showed that exoskeleton-based kinematics correlated to clinical outcome measures (30). More recently, Agrafiotis et al. analyzed the RMK data and developed predictive models of the clinical outcomes with the aim to remove inter- and intra-rater variability and reduce the sample size in stroke clinical trials (31). Even though the literature on clinical predictors after ulRT is well-established (32–36), only Duret et al. analyzed the RMK data with the aim to predict the upper limb recovery at the end of ulRT (37). However, the results obtained on 46 subacute stroke subjects evidenced that selected RMK parameters, calculated from the overall trajectory, do not predict the total upper limb Fugl-Meyer Assessment scores at the end of the treatment (37).

Nevertheless, considering the importance of evidence-based practice in stroke rehabilitation (38), the identification of patients who may benefit more from a robotic treatment (34) is needed. Especially, the pre-treatment motor status of subject should be analyzed in detail, considering the recent findings on the time course of motor recovery and the variations of the workspace exploration skills of a patient during ulRT (20, 39). In particular, movements characterized by elbow extension and shoulder flexion and by the abduction and external rotation of the shoulder are the most demanding to be executed by stroke subjects (40, 41) since these are against the abnormal flexor strategy. For these reasons, the recovery of these movements should be monitored as post-rehabilitation outcomes.

The aim of this study was to calculate a set of kinematic parameters from RMK data and to identify reliable predictors of upper-limb motor performance following ulRT in subacute stroke subjects. Specifically, the ability to execute point-to-point reaching movements in different directions has been considered as representative of motor impairment and of motor synergies in stroke survivors. Therefore, we hypothesized that the analysis of pre-treatment kinematic parameters would allow us to find predictors of upper limb recovery and, thus, to identify individuals who can benefit more from ulRT.

## Materials and Methods

An observational retrospective study was carried out on stroke subjects who had conducted ulRT in addition to the conventional therapy. This secondary analysis considered the RMK data for assessing the time course of motor recovery during ulRT (19). The data covered by this paper were acquired and processed by the IRCCS San Raffaele Roma (Rome, Italy).

### Selection of Patients

The study was conducted on a database of 271 inpatients who underwent ulRT with the planar end-effector InMotion 2.0 robot (Bionik Laboratories, Watertown, MA, USA) at the IRCCS San Raffaele Roma (Italy) between January 2011 and December 2017. Data were selected from patients who satisfied the following inclusion criteria: age between 18 and 80 years; first event of unilateral hemiparetic stroke; subacute phase (ulRT started within 30 ± 7 days post-stroke); upper limb Chedoke-McMaster scores between 2 and 5; Motricity Index affected Upper Limb <100; and ulRT for 20 sessions. The exclusion criteria were the following: bilateral impairment; chronic phase; ulRT for less than 20 sessions; interruption of the ulRT for more than 3 consecutive days; the presence of other severe medical conditions; and incomplete data in the database.

### Rehabilitative Protocol

**Figure 1:**
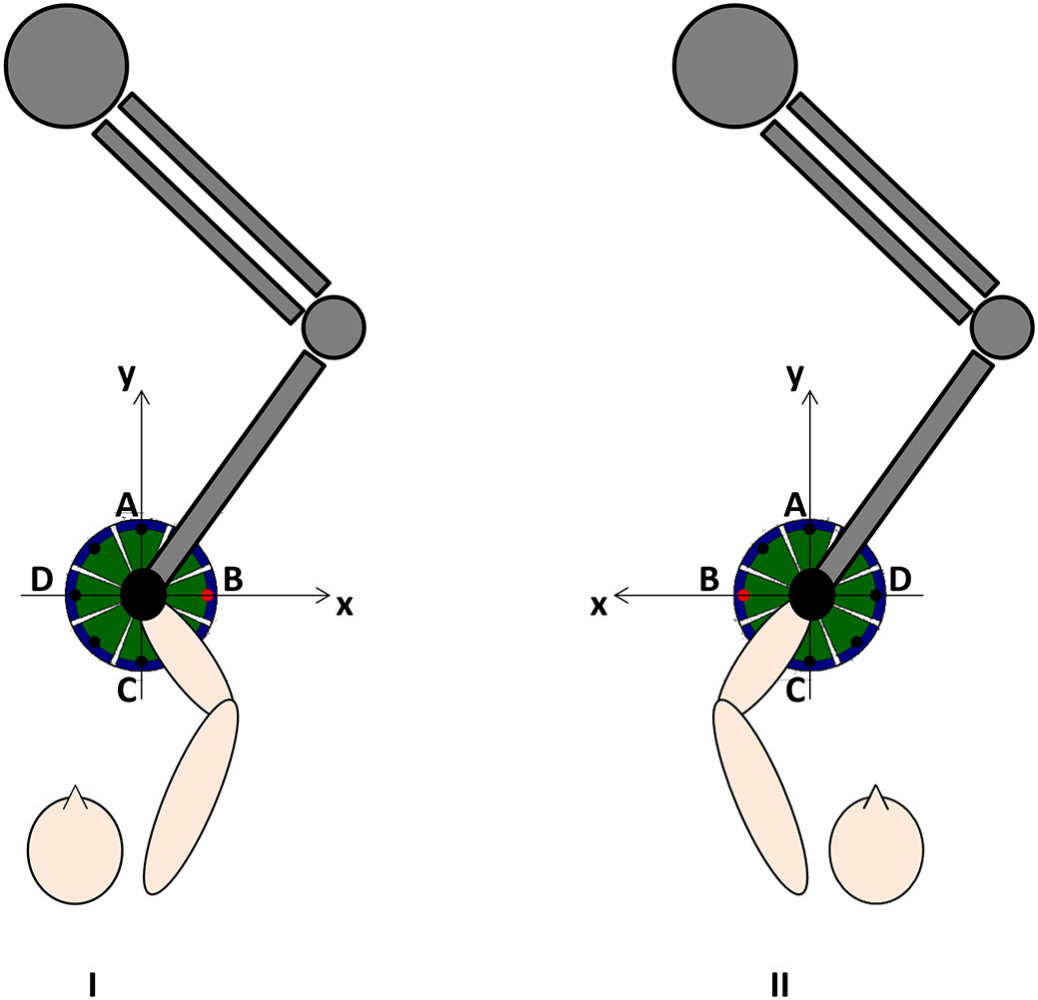
Experimental setup and reference system in case of right (I) or left (II) affected limb.

All subjects conducted 20 sessions (5 times/week) of InMotion2-based ulRT with an “assist as needed” strategy. The InMotion 2.0 device is an integrated system for interactive upper limb motor training and the simultaneous kinematic data registry (42). Each session of treatment lasted 45 minutes and consisted of the execution of a sequence of point-to-point reaching movements in the horizontal plane (16, 34). Each task involved the training of different muscle synergies, moving the end-effector from a central target to 8 peripheral targets, equally spaced on a 0.14 m radius circumference and vice versa (Figure 1). Visual biofeedback was delivered from a monitor placed in front of the subject. In addition, the subjects underwent conventional physiotherapy sessions according to the standardized rehabilitation protocol for subacute stroke patients: assisted stretching, shoulder and arm exercises, and functional reaching tasks. Detailed and relevant information on ulRT is available in the previous paper of the authors (20).

### Data Extraction

The demographic and clinical data have been extracted from the electronic medical records, such as age, gender, affected side, time since stroke, and etiology. The clinical and kinematic assessments were registered at the beginning (T1) and at the end (T2) of the ulRT. The privacy of patient was preserved by identifying each record in the database by means of a unique alphanumeric code.

The clinical outcomes are the following: modified Barthel Index (mBI) (4), Motricity Index of the affected Upper Limb (MI_UL_) (43), and the Motricity Index sub-items assessing the elbow flexion (MI_ELBOW_) and the shoulder abduction (MI_SHOULDER_). The item related to the pinch grip was not considered in this study because the InMotion2-based ulRT typically involves the elbow and shoulder joints.

The kinematic parameters were calculated from the trajectories recorded by the robot at 200 Hz, as detailed in the previous study of the authors (20). Specifically, the end-effector trajectory has been expressed with respect to a reference system consistent with the lesion side (Figure 1) and the following kinematic parameters have been calculated for each trajectory from the central target to the peripheral ones (directions of movement A, B, C, and D): Movement Path Error in centimeters (MPE); mean Movement Speed in centimeters/second (MS); and the number of Peaks Speed (nPS). The MPE is the mean absolute value of the minimum distance of each point of the actual path traveled by the subject from the ideal one (i.e., the straight line connecting the targets): the value is 0 if the trajectory lies exactly on a straight line connecting the targets. The MPE means how much the trajectory is far from the ideal straight line. The MS is the mean value of the resultant velocities in the plane where the trajectory lies. The nPS is defined as the number of peaks of the resultant velocity and it is a metric used for assessing the smoothness of the movement: low nPS values derive from few accelerations and decelerations, i.e., smooth movement.

The kinematic parameters computed in this study describe functional abilities and are in the “body function and structure” ICF domain as described by Tran et al. (24). They are considered as “performance metrics” for assessing the quality of the movement by assuming that the physiological reaching movements are straight, fairly quick, and smoothed (23, 29). Since the reference system is consistent with the lesion side (Figure 1), the directions of movement corresponded to the following major anatomical joint movements: A (elbow extension and shoulder flexion), B (abduction and external rotation of the shoulder), C (elbow flexion and shoulder extension), and D (adduction and internal rotation of the shoulder). Therefore, the ability to execute point-to-point reaching movements in different directions described by the kinematic parameters has been considered as representative of different synergies involved in the execution of the reaching tasks (51).

### Ethical Considerations

Since March 2012, the Italian Data Protection Authority (Garante per la protezione dei dati personali) declared that IRCCS (Istituto di Ricovero e Cura a Carattere Scientifico - Institute for scientific research and healthcare) are authorized to perform retrospective studies without the approval of the local Ethical Committee, and mandatory formal communication is sufficient. Such communication relative to this study was registered by the Ethical Committee of the IRCCS San Raffaele Roma on February 22, 2017 (code number: 06/17).

### Data Analysis and Statistical Analysis

The statistical analyses were performed on SPSS, Version 27.0 (SPSS Inc., Chicago, IL, USA, 2020). Descriptive statistics were computed to appropriately explain the characteristics of the sample. Data are represented as frequency (with the relative percentage), mean value with Standard Deviation (SD), and median value with Interquartile range (IQR) for the categorical, continuous, and ordinal variables, respectively. The Kolmogorov–Smirnov test with the Lilliefors correction was used to evaluate the normality of distribution. The statistically significant difference between T1 and T2 was assessed with paired *t*-test if the data were normally distributed, while Wilcoxon signed-rank test for other comparisons.

The regression analysis was applied for assessing the relationship between a dependent variable and a set of independent variables. The following analyses were conducted:

- Dependent variable: MI_ELBOW_ at T2. Independent variables: age, kinematic parameters at T1.
- Dependent variable: MI_SHOULDER_ at T2. Independent variables: age, kinematic parameters at T1.
- Dependent variable: MI_UL_ at T2. Independent variables: age, kinematic parameters at T1.
- Dependent variable: MPE A at T2. Independent variables: kinematic parameters at T1.
- Dependent variable: MPE B at T2. Independent variables: kinematic parameters at T1.
- Dependent variable: MS A at T2. Independent variables: kinematic parameters at T1.
- Dependent variable: MS B at T2. Independent variables: kinematic parameters at T1.
- Dependent variable: nPS A at T2. Independent variables: kinematic parameters at T1.
- Dependent variable: nPS B at T2. Independent variables: kinematic parameters at T1.

The MI_ELBOW_ and MI_SHOULDER_ are categorical variables composed of six classes, as defined by Wade (43). The MI_UL_ has been transformed into a categorical variable, by grouping the possible MI_UL_ values into the following six classes: class_0_ = 1–27; class_1_ = 29–40; class_2_ = 41–54; class_3_ = 55–66; class_4_ = 67–77; and class_5_ = 78–100.

The choice of considering, as dependent variables, the kinematic parameters calculated from the trajectories executed in direction A and B, was made taking into account that the motor tasks in these directions were the most challenging after stroke, as confirmed by the literature (20, 44).

In the regression analysis, the general linear model or the Generalized Linear Model (GLM) has been applied in the case of dependent variables with normal distribution or with no-normal distribution, respectively. Specifically, in the case of GLM, the following models have been used: Poisson model with a log link function, for categorical variables; Gamma model with a log link function, or Linear link identity for continuous variables; and multinominal cumulative logit for ordinal variables. The Pearson's χ^2^ and the deviance statistics were evaluated to assess the model's goodness of fit. The partial slope β was reported to measure the influences of each predictor. All tests were considered significant at a *p* < 0.05.

## Results

**Figure 2:**
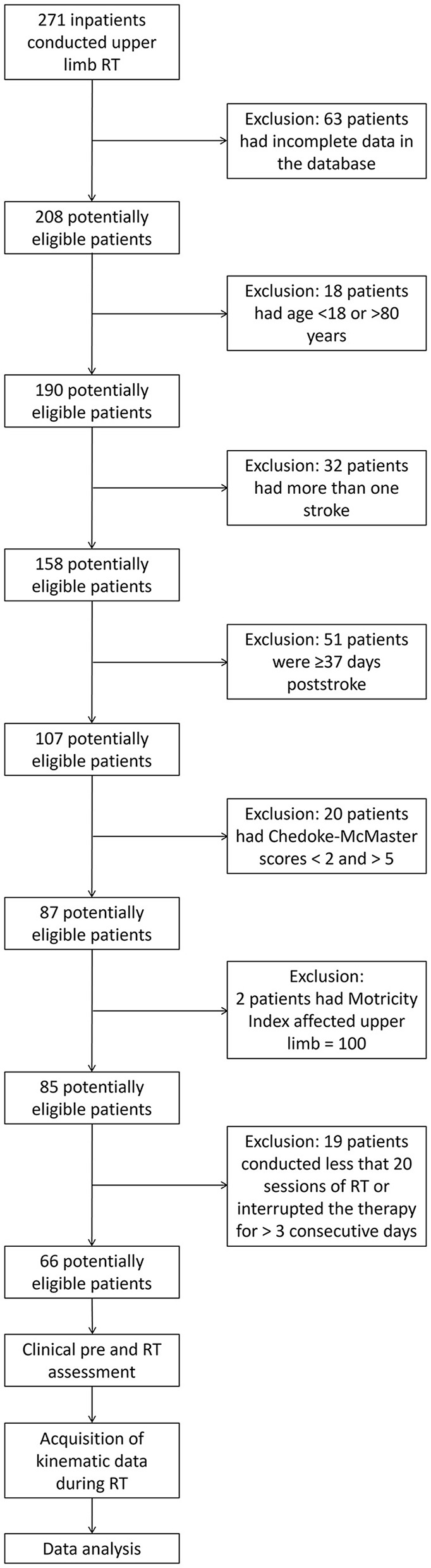
Consort diagram.

Starting from a database of 271 inpatients, 66 subacute stroke subjects satisfied the inclusion criteria and were included in the study (Figure 2). The mean age was 64.97 years (SD 12.75 years); 44 (66.7%) patients were male; and 39 (59.1%) subjects had the right upper limb impairment. Table 1 shows the demographic characteristics of the sample at baseline, the clinical scores (mBI, MI_ELBOW_, MI_SHOULDER_, and MI_UL_), and the kinematic parameters (MPE, MS, and nPS: directions A, B, C, and D) calculated at T1 and T2. At the end of ulRT, all clinical outcomes significantly improved (*p* < 0.05). The kinematic outcomes registered significant changes between T1 and T2 in all parameters except the MPE calculated from the trajectories executed in direction B (*p* = 0.159).

**Table 1:** Summary of the sample characteristics before (T1) and after (T2) the upper-limb robot-assisted therapy (ulRT).

	***N* (%)**	**T1**	**T2**	***p*-value**
Age (years)		64.97 ± 12.75		
Gender, male/female	44 (66.7)/22 (33.3)			
Side, right/left	39 (59.1)/27 (40.9)			
Time since stroke (days)		15.27 ± 18.07		
Etiology, ischemic/hemorrhagic	47 (71.2)/19 (28.8)			
mBI		26.0 (14.75–40.25)	79.5 (64.25–92.25)	<0.001^*^
MI_ELBOW_		14.0 (9.0–19.0) 14.2 ± 9.6	25.0 (17.75–33.0) 23.8 ± 10.3	<0.001^*^
MI_SHOULDER_		14.0 (9.0–19.0) 14.0 ± 9.1	25.0 (17.75–33.0) 23.5 ± 9.8	<0.001^*^
MI_UL_		42.0 (19.0–62.0) 42.6 ± 27.8	77.0 (47.25–93.0) 69.4 ± 30.2	<0.001^*^
MPE A (m)		0.021 (0.012–0.043)	0.014 (0.010–0.028)	0.004^*^
MPE B (m)		0.016 (0.007–0.026)	0.013 (0.007–0.022)	0.159^*^
MPE C (m)		0.017 (0.012–0.029)	0.010 (0.01–0.02)	0.002^*^
MPE D (m)		0.017 (0.011–0.030)	0.010 (0.01–0.02)	0.002^*^
MS A (m/s)		0.061 ± 0.043	0.085 ± 0.040	<0.001+
MS B (m/s)		0.074 ± 0.049	0.112 ± 0.054	<0.001+
MS C (m/s)		0.058 (0.031–0.095)	0.10 (0.08–0.14)	<0.001^*^
MS D (m/s)		0.074 ± 0.050	0.108 ± 0.048	<0.001+
nPS A		6.0 (4.0–9.0)	2.0 (1.0–3.0)	<0.001^*^
nPS B		4.0 (2.0–7.0)	2.0 (1.0–3.0)	<0.001^*^
nPS C		4.0 (2.0–8.0)	2.0 (1.0–4.0)	<0.001^*^
nPS D		4.0 (2.0–7.0)	1.5 (1.0–3.0)	<0.001^*^

*Data are shown as mean ± SD or median (interquartile range [IQR])*.

* *Wilcoxon signed-rank tests*.

+ *Paired t-test. mBI, modified Barthel Index; MI_ELBOW_, Motricity Index affected elbow flexion; MI_SHOULDER_, Motricity Index affected shoulder abduction; MI_UL_, Motricity Index affected Upper Limb; MPE, Movement Path Error; MS, mean Movement Speed; nPS, number of Peaks Speed. T1: before the ulRT; T2: after the ulRT. Directions of movement: A, B, C, D*.

Table 2 presents the results of the regression analysis of factors associated with the upper limb motor impairment: the model used for the analysis was the GLM with multinomial cumulative logit since the dependent variables (MI_ELBOW_, MI_SHOULDER_, and MI_UL_) were ordinal. The age, the path errors (MPEs in directions A, C, and D), and the speed (MS in direction B) at T1 were significant predictors of MI_ELBOW_, MI_SHOULDER_, and MI_UL_ at T2. Specifically, older subjects were less likely to increase the Motricity Index of the affected upper limb: all subjects improved their level of impairment at T2 but for each year of age, the probabilities to increase of one class in the MI_ELBOW_, MI_SHOULDER_, and MI_UL_ diminished by 7.0, 5.8, and 7.3%, respectively. The MPEs calculated from the trajectories executed in direction A and C were significant positive predictors of MI_ELBOW_, MI_SHOULDER_, and MI_UL_: the less accurate the trajectories were at baseline (i.e., high MPE values), the more the MI_ELBOW_, MI_SHOULDER_, and MI_UL_ increased at the end of the treatment. Conversely, the MPE (direction D) and the MS (direction B) were negative prognostic factors for motor impairment at the end of the ulRT: less accurate trajectories in direction D and quick movements in direction B at T1 negatively interfere with the increase of MI_ELBOW_, MI_SHOULDER_, and MI_UL_ at T2. The remaining independent variables did not significantly contribute in predicting the clinical assessment of motor impairment at T2.

**Table 2:** Results of the regression analysis of factors associated with the upper limb motor impairment at the end of ulRT.

***Modeled using GLM with Multinominal cumulative logit***	**MI**_**ELBOW**_ **T2**					**MI**_**SHOULDER**_ **T2**					**MI**_**UL**_ **T2**				
	**β**	**SE**	**OR**	**95%CI**		**β**	**SE**	**OR**	**95%CI**		**β**	**SE**	**OR**	**95%CI**	
Age	−0.072*	0.023	0.930	0.890	0.972	−0.059*	0.0225	0.942	0.902	0.985	−0.076*	0.0237	0.927	0.885	0.971
MPE A T1	0.003*	0.001	1.003	1.001	1.006	0.004*	0.0014	1.004	1.001	1.007	0.003*	0.0013	1.003	1.001	1.005
MPE B T1	0.001	0.002	1.001	0.998	1.004	0.002	0.0013	1.002	0.999	1.004	0.001	0.0013	1.001	0.998	1.003
MPE C T1	0.006*	0.002	1.006	1.003	1.009	0.006*	0.0015	1.006	1.003	1.009	0.005*	0.0015	1.005	1.002	1.008
MPE D T1	−0.007*	0.002	0.993	0.989	0.997	−0.007*	0.0021	0.993	0.989	0.997	−0.006*	0.0022	0.994	0.989	0.998
MS A T1	−0.001	0.001	0.999	0.998	1.001	−0.001	0.0007	0.999	0.998	1.001	−0.001	0.0008	0.999	0.998	1.001
MS B T1	−0.002*	0.001	0.998	0.996	0.999	−0.002	0.0010	0.998	0.996	1.000	−0.002	0.0011	0.998	0.996	1.000
MS C T1	0.002	0.001	1.002	0.999	1.004	0.001	0.0011	1.001	0.998	1.003	0.001	0.0012	1.001	0.999	1.003
MS D T1	0.001	0.001	1.001	0.999	1.003	0.002	0.0008	1.002	1.000	1.003	0.001	0.0009	1.001	0.999	1.003
nPS A T1	0.012	0.094	1.013	0.841	1.218	0.018	0.0970	1.018	0.841	1.231	−0.011	0.0967	0.989	0.818	1.195
nPS B T1	0.124	0.097	1.132	0.9360	1.370	0.021	0.0902	1.021	0.855	1.218	0.125	0.0965	1.133	0.938	1.369
nPS C T1	−0.012	0.060	0.988	0.879	1.111	0.002	0.0601	1.002	0.890	1.127	−0.022	0.0606	0.979	0.869	1.102
nPS D T1	0.067	0.102	1.069	0.875	1.306	0.154	0.1086	1.167	0.943	1.443	0.132	0.1081	1.141	0.923	1.410
**Threshold**															
Class score 1	0.049	1.6914	1.050	0.038	28.911	2.269	1.7729	9.673	0.300	312.33	−0.948	1.7491	0.387	0.013	11.940
Class score 2	−1.010	1.6645	0.364	0.014	9.513	1.155	1.7232	3.173	0.108	92.946	−1.981	1.7656	0.138	0.004	4.391
Class score 3	−1.829	1.6706	0.161	0.006	4.243	−0.117	1.7146	0.889	0.031	25.617	−2.649	1.7840	0.071	0.002	2.335
Class score 4	−2.560	1.6788	0.077	0.003	2.077	−1.173	1.7071	0.309	0.011	8.782	−3.254	1.7901	0.039	0.001	1.290
Class score 5	−3.987*	1.7098	0.019	0.001	0.529	−2.334	1.6961	0.097	0.003	2.691	−3.900*	1.7932	0.020	0.001	0.680
Deviance	0.552					0.568					0.571				
Pearson's Chi Square/gdl	1.998					1.163					1.273				
AIC	208.270					213.251					214.105				
Likelihood ratio	33.134*					32.74*					28.329*				

*MI_ELBOW_, Motricity Index affected elbow flexion; MI_SHOULDER_, Motricity Index affected shoulder abduction; MI_UL_, Motricity Index affected Upper Limb; MPE, Movement Path Error; MS, mean Movement Speed; nPS, number of Peaks Speed. T1: before the ulRT; T2: after the ulRT. Directions of movement: A, B, C, and D*.

* *p < 0.05*.

The regression analysis of MPE and MS was executed with the GLM with the Gamma distribution (with a log link function) and with Linear link identity, respectively. Since the response of the nPS is a count, the nPS A and nPS B respected the assumptions to perform the GLM with Poisson distribution (with a log link function). The histograms of MPE, MS, and nPS and the corresponding distributions are showed in Appendix.

Table 3 (I) shows the results of the GLM for MPE direction A at T2. The MPE direction B was not considered as a dependent variable because it did not significantly change between T1 and T2 (as shown in Table 1). The analysis revealed that the nPS direction A was a significant protective factor of MPE direction A (odds ratio [*OR*] = 1.088; 95% *CI* = 1.020–1.159). Expressly, having one peak more in the resultant velocity (direction A) at T1 increases 8.8% of the probability to have a higher path error (i.e., MPE) in executing point-to-point movements in direction A at T2.

**Table 3:** Results of the regression analysis of factors associated with: (I) the movement path error (MPE) direction A at the end of ulRT; (II) the movement speed (MS) direction A and direction B at the end of ulRT.

**I)**	***Modeled using GLM with Gamma link log***	**MPE A T2**							
		**β**	**SE**	**OR**	**95%CI**				
	MS A T1	−0.894	2.554	0.409	0.003	61.073			
	MS B T1	−0.315	3.161	0.729	0.001	35.790			
	MS C T1	2.630	3.811	13.871	0.008	24354.400			
	MS D T1	1.309	2.818	3.704	0.015	928.756			
	nPS A T1	0.084*	0.033	1.088	1.020	1.159			
	nPS B T1	−0.003	0.029	0.997	0.942	1.056			
	nPS C T1	0.023	0.021	1.023	0.982	1.066			
	nPS D T1	0.025	0.027	1.025	0.973	1.081			
	Intercept	−4.844*	0.418	0.008	0.003	0.018			
	Deviance	0.561							
	Pearson's Chi Square/gdl	0.556							
	AIC	−379.092							
	Likelihood ratio	10.313*							
**II)**	***Modeled using GLM with linear link identity***	**MS A T2**				**MS B T2**			
		**β**	**SE**	**95%CI**		**β**	**SE**	**95%CI**	
	MPE A T1	−0.349	0.198	−0.738	0.039	−0.270	0.274	0.447	1.305
	MPE B T1	0.158	0.213	−0.260	0.576	0.409	0.294	0.846	2.681
	MPE C T1	−0.478*	0.241	−0.949	−0.006	−0.366	0.332	0.362	1.329
	MPE D T1	0.461	0.309	−0.144	1.066	0.219	0.426	0.540	2.868
	nPS A T1	0.003	0.002	0.999	1.006	0.003	0.002	−0.002	0.007
	nPS B T1	−0.001	0.002	0.995	1.002	−0.006*	0.002	−0.010	−0.001
	nPS C T1	−0.001	0.001	0.997	1.001	−0.001	0.001	−0.003	0.002
	nPS D T1	−0.002	0.002	0.994	1.001	−0.001	0.002	−0.006	0.003
	Intercept	0.101*	0.013	0.076	0.126	0.137*	0.017	0.103	0.171
	Deviance	0.001				0.003			
	Pearson's Chi Square/gdl	0.001				0.003			
	AIC	−236.621				−194.145			
	Likelihood ratio	18.853*				17.144*			

*MPE, Movement Path Error; MS, mean Movement Speed; nPS, number of Peaks Speed. T1: before the ulRT; T2: after the ulRT. Directions of movement: A, B, C, and D*.

* *p < 0.05*.

Table 3 (II) depicts the predictors of MS direction A and direction B at T2. The results show that the MPE direction C and the nPS direction B were negative prognostic factor for MS direction A and B, respectively. For each one-unit increase in MPE direction C, the expected value of the dependent variable (MS direction A) increases by β = −0.478, assuming all other variables constant. Therefore, the greater the MPE direction C at baseline, the smaller the MS direction A at the end of the treatment. For each one-unit increase in nPS B, the expected value of MS direction B decreases at T2 (β = −0.006): the more fluid movements are performed toward B (against the pathological pattern) at T1, the more rapid movements are performed toward B at T2.

Table 4 presents the outcomes of the GLM with Poisson distribution (with a log link function) with the nPS direction A and direction B at T2 as dependent variables. The MPE direction A at baseline was found to be a significant predictor of both dependent variables (nPS direction A: *OR* = 1.001, 95% *CI* = 1.001–1.002; nPS direction B: *OR* = 1.001, 95% *CI* = 1.000–1.001). Thus, the greater the MPE direction A at T1, the greater the nPS direction A and direction B at T2. On the other hand, having higher MS direction B at baseline decreases the probability to have high nPS in the same direction at the end of ulRT (*OR* = 0.999, 95% *CI* = 0.999–1.000): the greater the MS direction B the smaller the nPS in the same direction.

**Table 4:** Results of the regression analysis of factors associated with the number of peaks speed direction A and direction B at the end of ulRT.

***Modeled using GLM with Poisson link log***	**nPS A T2**					**nPS B T2**				
	**β**	**SE**	**OR**	**95%CI**		**β**	**SE**	**OR**	**95%CI**	
MPE A T1	0.001*	0.0003	1.001	1.001	1.002	0.001*	0.0003	1.001	1.001	1.002
MPE B T1	0.000	0.0003	1.000	0.999	1.000	−0.083	0.0003	1.000	0.999	1.001
MPE C T1	0.000	0.0003	1.000	1.000	1.001	0.001	0.0003	1.001	1.000	1.001
MPE D T1	0.000	0.0004	1.000	0.999	1.001	0.000	0.0004	1.000	0.999	1.000
MS A T1	0.000	0.0002	1.000	0.999	1.000	−0.001*	0.0003	0.999	0.998	0.999
MS B T1	0.000	0.0003	1.000	0.999	1.000	−0.001*	0.0003	0.999	0.998	0.999
MS C T1	0.000	0.0004	1.000	0.999	1.000	0.081	0.0004	1.000	0.999	1.001
MS D T1	0.000	0.0002	1.000	1.000	1.001	0.000	0.0003	1.000	1.000	1.001
Intercept	1.277*	0.199	3.585	2.429	5.292	1.298*	0.221	3.663	2.375	5.650
Deviance	1.115					1.119				
Pearson's Chi Square/gdl	1.128					1.344				
AIC	268.739					255.541				
Likelihood ratio	51.871*					43.666*				

*MPE, Movement Path Error; MS, mean Movement Speed; nPS, number of Peaks Speed. T1: before the ulRT; T2: after the ulRT. Directions of movement: A, B, C, and D*.

* *p < 0.05*.

## Discussion

This observational retrospective study analyzed the upper limb kinematics and the clinical characteristics of subacute stroke subjects, who received ulRT, to find potential inferences on the degree of impairment with motor outcomes at the end of the treatment. To this aim, data from 66 subjects were analyzed by GLMs to explore all potential relations between the dependent variables and every independent variable as predictive biomarkers. Although the literature on the clinical predictors after ulRT is well-established (32–36), a limited number of studies aimed to find predictors from data registered by a robot for rehabilitation (31, 37): however, the published studies aimed to predict the clinical outcomes and calculated the RMK features from complex trajectories composed by a set of movements having different directions in the workplace, thus did not discriminate the performance in executing movements with different directions. To the best of our knowledge, this is the first attempt at a multidirectional analysis of RMK data to find potential predictive biomarkers of motor outcomes after an intensive rehabilitation protocol that combined ulRT with conventional rehabilitation.

In our study, all the clinical and RMK outcomes (except the trajectories executed in direction B) significantly improved at the end of the treatment, in accordance with studies on the efficacy of ulRT in stroke survivors (9, 16). The obtained improvement could depend on the high dose physical rehabilitation, since the patients conducted a highly intensive ulRT (20 sessions, 5 times/week), considering the literature on the topic (5).

The results obtained from the regression analysis of upper limb motor impairment showed that age was a significant negative prognostic factor, in agreement with the literature on predictors of upper limb recovery following stroke (45). Furthermore, a subset of kinematic parameters calculated at baseline evidenced significant effects on motor impairment after ulRT, thus suggesting a correlation between upper limb kinematics and clinical outcomes. The trajectory accuracy is a significant positive predictor of upper limb recovery, and the analysis of the MPEs at baseline may suggest the pattern of motor recovery at the end of ulRT. Adduction and internal rotation movements of the shoulder are known as the typical abnormal strategy of stroke survivors (28, 40). Consolidated literature, in fact, described the stereotyped movement patterns characterized by simultaneous shoulder abduction and elbow flexion as flexor synergy (41). In our study, patients with good ability to perform these movements were more likely to recover upper limb motor function at the end of the treatment: as shown in Table 2, less accurate trajectories toward D and quick movements toward B at baseline negatively interfere with the increase of MI_ELBOW_, MI_SHOULDER_, and MI_UL_ at the end of ulRT. Conversely, the ability to perform trajectories characterized by flexion-extension movements of the elbow negatively affects motor recovery: subjects who executed less accurate and controlled elbow movements at baseline were more likely to recover upper limb functions at the end of the treatment. These results are in accordance with Dipietro et al. (41) who described the changes in the motor performance of the circle drawing task executed by chronic stroke subjects with the same robot, finding a correlation with the process of tuning of motor synergies that underlies stroke recovery. Our findings suggest that more severely compromised patients appear to have a better chance of recovery after ulRT. Indeed, it is worth to mention that this outcome could be the result of a ceiling effect of RMK measurements for the less severely damaged patients, as Agrafiotis et al. claimed (31).

Considering the evidence on the time course of kinematic parameters during the ulRT (28), the movements characterized by elbow extension and shoulder flexion (target A) and by the abduction and external rotation of the shoulder (target B) are the most difficult to be executed at the end of ulRT (20). The ability to perform good elbow extension and shoulder flexion movements after ulRT is significantly dependent on the ability to execute accurate and smooth elbow flexion-extension movements at baseline. Moreover, good control of movements toward B at the end of the treatment depends on the ability to perform accurate elbow extension movements at baseline. On the other hand, the smoothness and speed of abduction and external rotation movements of the shoulder are correlated to good levels of the same kinematic parameters at baseline. Subjects who did not present upper limb spastic co-contraction and abnormal motor synergies at T1 and, as a result, executed smooth and accurate movements toward A, had a higher probability to recover a more physiological motor control (i.e., direction A and B) characterized by high accuracy and smoothness. This outcome is in accordance with the literature on clinical aspects of upper limb motor impairment after stroke (46, 47). Specifically, Rohrer et al. (46) analyzed the movement smoothness changes using RMK features from the same robot and found a significant difference between the subacute and chronic patients, and a moderate correlation to the Fugl-Meyer Assessment score. In addition, the smoothness and submovement changes in chronic stroke patients have been analyzed by Dipietro et al. (47) who found that by the end of the training movements became smoother and that it could be explained by changes in increasingly overlapping submovements, which became fewer, longer, and faster during recovery. These outcomes suggested that recovery starts first by regaining the ability to generate submovements and then, over a longer time-period, by reacquiring the means to combine them.

The kinematic parameters calculated from the trajectories toward the target C can be representative of upper limb spastic co-contraction (48, 49). Therefore, the less accurate the trajectory in C (meaning that the flexion of the elbow is not well-controlled), the lower the speed in A (stimulating the extension of the elbow), which could represent the spasticity level. Similarly, the smoothness (nPS direction C) can be a predictor of accurate trajectories at T2 toward B. These outcomes are in accordance with the literature on upper limb kinematics, showing that the movements of stroke patients are characterized by slow and segmented trajectories (50, 51), and that motor recovery increases movement smoothness and decrease the number of velocity peaks (48). The kinematic parameters calculated from the trajectories toward target D (toward the hemiparetic side) are not predictive of any kinematic parameter at the end of ulRT.

This study presented some limitations that deserve to be discussed. The retrospective design of the research is associated with the presence of potential confounding factors, with the limited number of subjects, and with the absence of an assessment of spasticity. However, environmental influences are minimized considering that the recruited subjects underwent additional conventional physiotherapy according to the standard rehabilitative protocol for subacute stroke patients. In future studies, an assessment of spasticity, joint sensory and proprioception, and a description of any flexion synergy should be included in the study design. Another limitation is that the motion kinematics was assessed from RMK data, which could have biased the results. Moreover, the study considered a planar end-effector robot, while robotic exoskeletons for ulRT are available. The future research agenda should consider longitudinal large studies, involving different types of robots for rehabilitation, and could assess the motion kinematics with motion capture systems, such as stereophotogrammetry or inertial sensors (22).

Nevertheless, the obtained results showed that the ability to execute point-to-point reaching movement in different directions can be considered as representative of motor recovery and that a multidirectional analysis of pre-treatment RMK data may help to identify subjects who could benefit more from ulRT. In fact, although the studies on ulRT evidenced that robotic training is effective in stroke patients (6), the literature on the impact of characteristics and abilities of patients on the motor performance outcomes is not consistent.

## Conclusions

The multidirectional analysis of pre-treatment RMK data allows predicting motor recovery after ulRT in subacute stroke survivors. Specifically, kinematic parameters calculated at baseline can help the clinicians in defining the rehabilitative program, tailoring the ulRT to the characteristics and abilities of patients at baseline. Specifically, an additional ulRT training in the elbow extension and shoulder flexion and in the abduction and external rotation of the shoulder may help reduce the upper limb flexion synergies and could be a good rehabilitation strategy in the subjects with negative predictors. The end-effector ulRT could be considered more effective for severely compromised patients who appear to have a greater chance of recovery and reduce their impairment. On the other hand, less impaired stroke patients have a higher probability to recover a more physiological motor control characterized by point-to-point reaching movements with high accuracy and smoothness.

## Data Availability Statement

The raw data supporting the conclusions of this article will be made available by the authors, without undue reservation.

## Ethics Statement

The studies involving human participants were reviewed and approved by IRCCS San Raffaele Roma. The patients/participants provided their written informed consent to participate in this study.

## Author Contributions

MG, MF, and FP have made substantial contributions to conception and design. SPo and AG participated in the enrolment phase and carried out the treatment. MG and SPo carried out the clinical and kinematic assessments. MG and SPr designed the algorithm for data analysis. MG, SPo, SPr, AG, FP, and MF participated in the study design and coordination and statistical analysis. SP and FP participated in the manuscript revisions. MG and MF gave the final approval of the version. All authors contributed to the article and approved the submitted version.

## Funding

This project was partially funded by the Ministry of Health (ricerca corrente), Italy.

## Conflict of Interest

The authors declare that the research was conducted in the absence of any commercial or financial relationships that could be construed as a potential conflict of interest.

## Publisher's Note

All claims expressed in this article are solely those of the authors and do not necessarily represent those of their affiliated organizations, or those of the publisher, the editors and the reviewers. Any product that may be evaluated in this article, or claim that may be made by its manufacturer, is not guaranteed or endorsed by the publisher.
