# Supplementary figures and images for: Retrospective Robot-Measured Upper Limb Kinematic Data From Stroke Patients Are Novel Biomarkers
Source: Front Neurol. 2021 Dec 21;12:803901. doi: 10.3389/fneur.2021.803901 (PMC8725786; doi:10.3389/fneur.2021.803901)

## Supplementary Material

### APPENDIX

Distributions of MPE A, MPE B, MS A, MS B, nPS A, and nPS B.

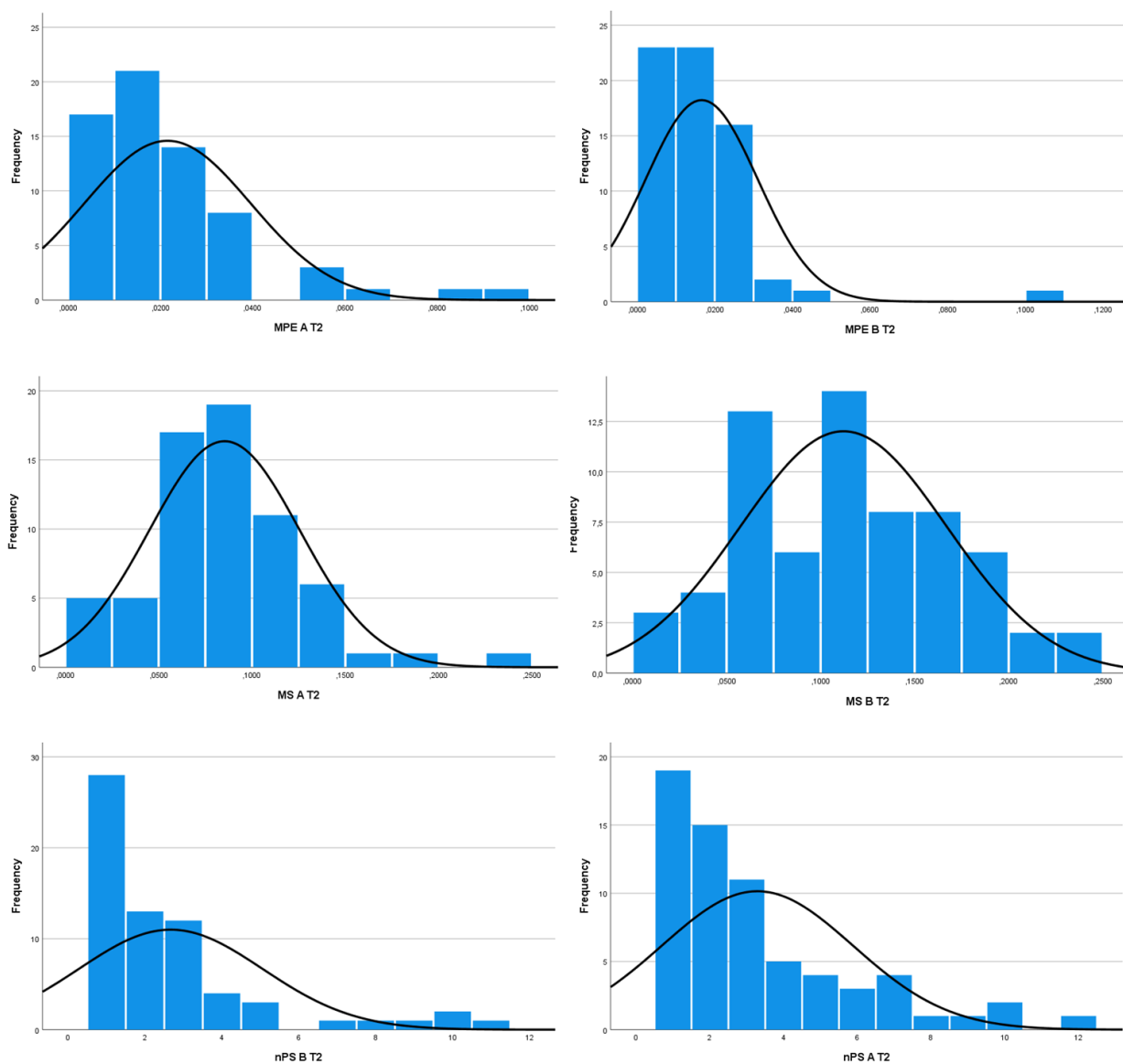

Supplement: Supplementary file 1 [file Data_Sheet_1.PDF]
